# Bond Dissociation Dynamics of Single Molecules on a Metal Surface

**DOI:** 10.1021/acsnano.4c17652

**Published:** 2025-03-05

**Authors:** Donato Civita, Matthew Timm, Jutta Schwarz, Stefan Hecht, Leonhard Grill

**Affiliations:** 1 Department of Physical Chemistry, University of Graz, Heinrichstraße 28, Graz 8010, Austria; 2 Department of Chemistry & Center for the Science of Materials Berlin, Humboldt University Berlin, Brook-Taylor-Str. 2, Berlin 12489, Germany

**Keywords:** scanning tunneling microscopy, silver surfaces, single-molecule chemistry, bond dissociation, molecular dynamics, organic molecules, carbon−halogen bond

## Abstract

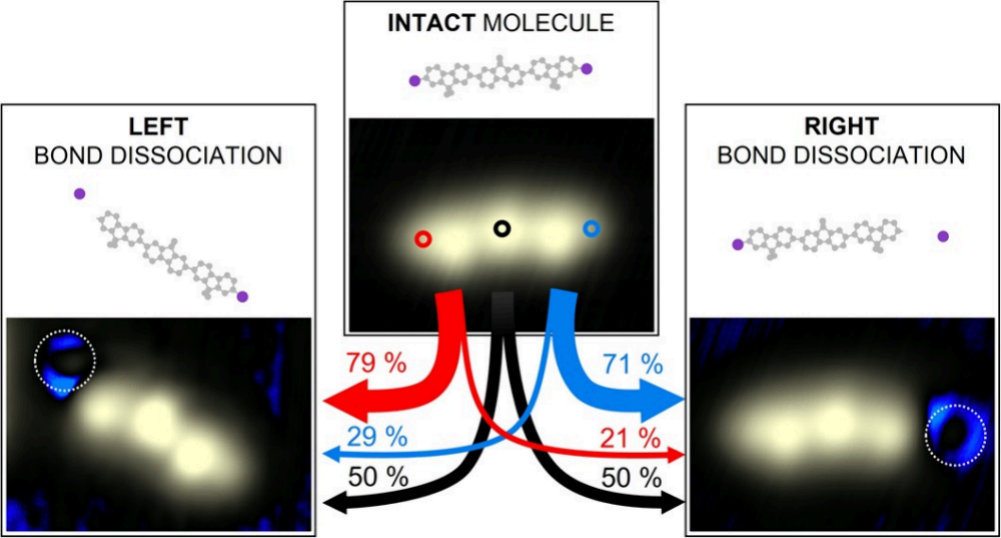

The breaking of an interatomic bond is at the heart of chemistry yet remains a challenge to be investigated. Molecules on metal surfaces exhibit defined positions and orientations and can be characterized by scanning tunneling microscopy that moreover is able to trigger bond breaking. Until now, the bond dissociation dynamics has been studied in small molecules but not in large ones with various degrees of freedom. Here, we dissociate bromine atoms from single dibromo-terfluorene molecules on Ag(111), identifying not only the displacement but also the rotation of each fragment. It turns out that the molecular excitation that causes dissociation is not locally confined. Instead, it can propagate through the molecule, and the dynamics of the resulting fragments is uncorrelated. The fragment binds to the nearest silver atom after dissociation and dissipates its energy in rotational motion. Our findings could be useful for the precise engineering of chemical reactions with prearranged precursor molecules.

The characterization of bond breaking dynamics is important to elucidate how the available energy is distributed from the parent to the product molecules. To characterize the dynamics, various parameters of a chemical reaction must be known: the energy and collision geometry of the reactants as well as the energy and angular distribution of the products. Detailed insights have been obtained with crossed molecular beam experiments in vacuum,^1,2^ which average over many possible reaction geometries, as the trajectory and geometrical arrangement of every single collision cannot be precisely controlled. This is fundamentally different in so-called surface-aligned reactions^3,4^ where the reactants are adsorbed in a defined geometry on a single-crystal surface.

Metal surfaces are of particular interest as they are fundamental in heterogeneous catalysis, where they lower the activation energy required for many chemical transformations ranging from addition to dissociation reactions.^5^ For detailed insights into the dynamics of bond dissociation, it is necessary to investigate the reaction at the single-molecule level on crystalline, atomically defined metal surfaces. Single bond dissociations have been triggered with voltage pulses from a scanning tunneling microscope (STM) tip.^6−12^ In particular, the STM has been used to study the cleavage of carbon–halogen bonds,^13−16^ which can be done chemo- and regioselectively within an individual molecule.^6−8^ Typically, only one bond is broken, but in rare cases a single-electron event can dissociate two bonds within a molecule, or a chain of molecules due to charge delocalization.^17−19^ For detailed insight into the dissociation process, small molecules are not helpful as different positions of the pulse affect the same parts of the compound.^20^ Additionally, it is also challenging to resolve their orientation on the surface. However, information on the orientation is crucial to describe how the energy is dissipated during the bond dissociation process in translational, vibrational, and rotational degrees of freedom of the molecular fragments.

We used molecules with an elongated chemical structure, i.e., dibromo-terfluorene (DBTF), to investigate the rotation of molecular fragments and to test the effect of the pulse position. They are composed of three fluorene moieties, constituting the elongated π-conjugated backbone of the molecule, and lateral dimethyl groups, which lift it from the substrate (Figure 1A).^21^ In our experiments, we selectively induce dissociation of one of the terminal Br substituents by using a pulse of tunneling electrons from the STM tip. Due to its rod-like shape, the molecule can be positioned on the surface with a defined orientation and can be imaged after debromination in its final position and orientation.

**Figure 1:**
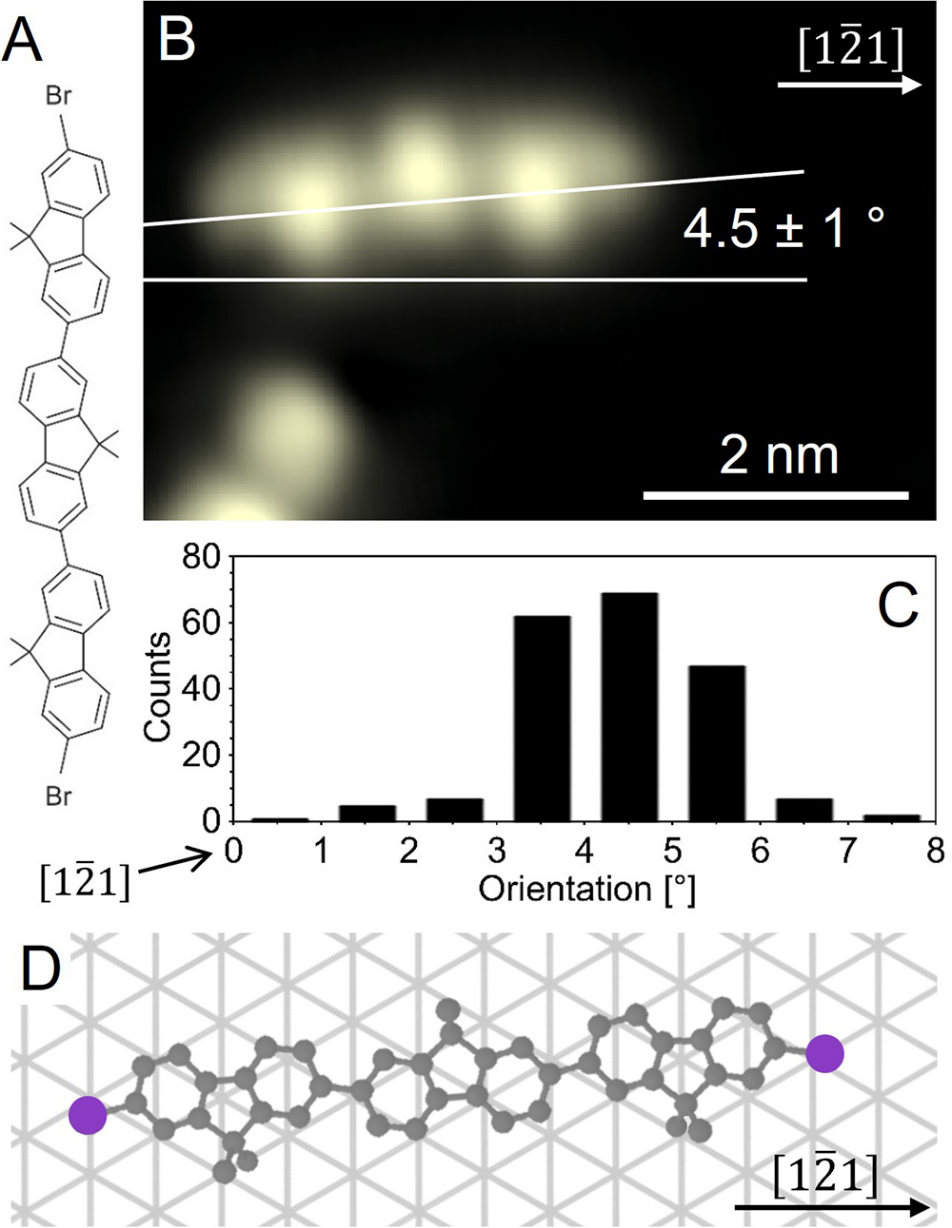
**Adsorption configuration of a DBTF molecule on Ag(111).** (A) Chemical structure of dibromo-terfluorene (DBTF). (B) STM image (0.6 V, 300 pA) of a single DBTF molecule on Ag(111). The orientation angle of the molecule with respect to the [12̅1] direction of the surface is indicated. (C) Histogram obtained from 199 measurements of the orientation of single DBTF molecules. The data are folded considering the 6-fold symmetry of the Ag(111) surface (Figure S3) and the symmetry of the molecule (Figure S4). In this histogram the [12̅1] symmetry direction of the Ag(111) surface is set at 0°, and positive angles are measured in the counterclockwise direction. (D) Scheme of the adsorption configuration of a single DBTF molecule on Ag(111), obtained from the analysis described in Figure S4. Gray circles represent carbon atoms and the violet circles bromine atoms, while the silver surface is plotted as a lattice.

## Results and Discussion

After extracting a single molecule from a molecular island (Figure S1) by lateral manipulation with the STM tip (Figure S2), the STM image of a single DBTF molecule (Figure 1B) shows three dominant lobes with 1.60 ± 0.03 Å apparent height, which originate from the dimethyl groups, while the Br substituents appear as shoulders at the termini, about 2.9 nm apart. The orientation of the DBTF molecules, determined from STM images recorded after bringing them as isolated entities to a flat terrace, is 4.5° ± 1° with respect to the [12̅1] high symmetry direction of the Ag(111) surface (Figure 1C–D). Note that adsorption at -4.5° is observed with the same abundance, which–due to the molecular symmetry–is equivalent to the +4.5° orientation, therefore the data were folded (details in Figures S3–S4). Moreover, with the help of bromine atoms as reference points, we were able to extract the precise position of the molecule with respect to the atomic lattice of the substrate (Figure S4), shown in Figure 1D.

### Electron-Induced Dissociation

**Figure 2:**
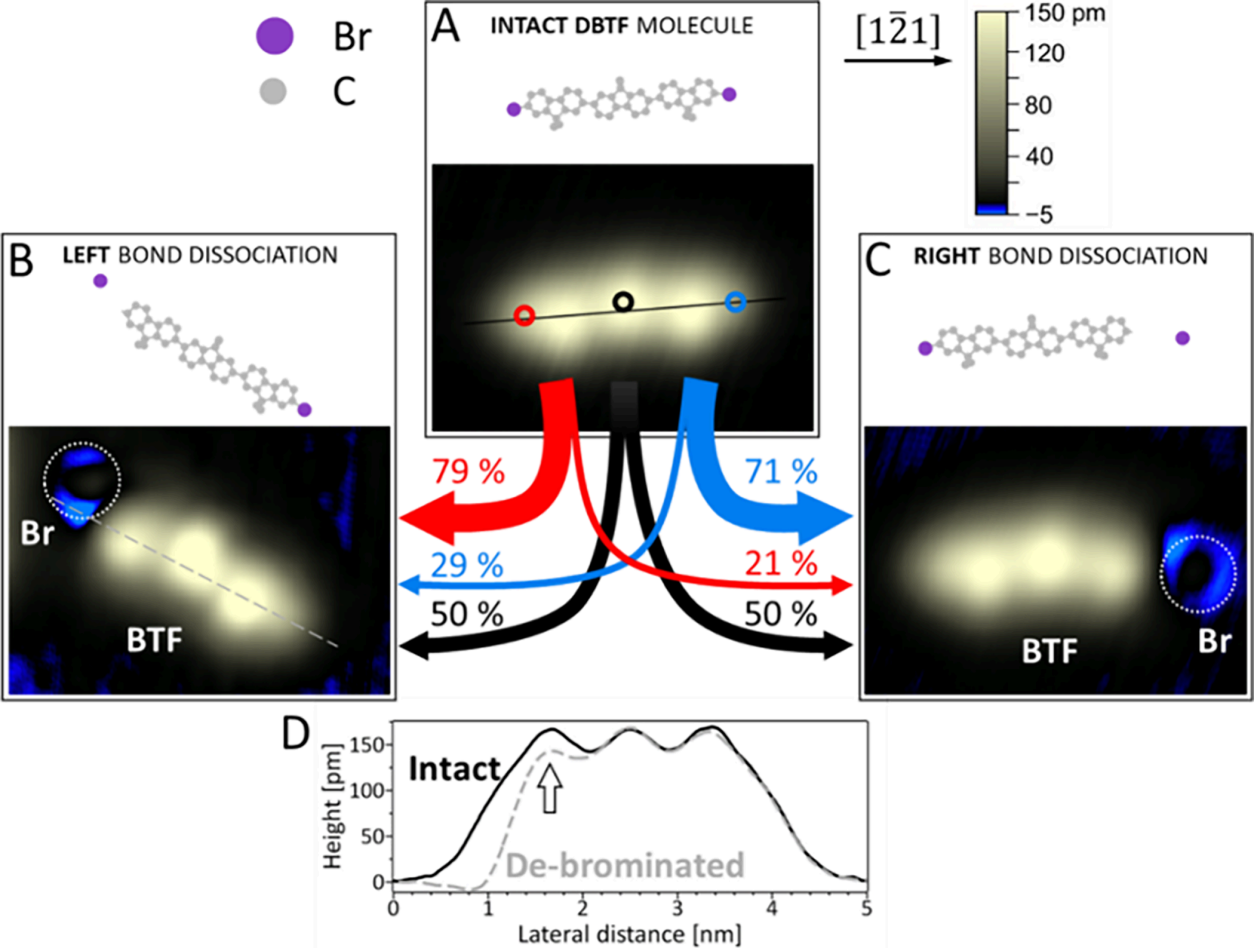
**Electron-induced dissociation of a Br atom from a DBTF molecule on Ag(111).** STM images (each 5.0 × 3.5 nm^2^ in size, 300 pA, 0.6 V) of single DBTF molecules on Ag(111) before (A) and after (B,C) Br dissociation with corresponding schemes above them (gray and violet circles represent C and Br atoms, respectively). The color scale is shown on the top-right corner, blue color indicates a depression below the black level of the substrate set at 0 pm. The blue “sombrero-shaped” feature in the dashed circle (B,C) is the Br atom. All images are oriented with the [12̅1] symmetry direction of the surface set horizontally, as indicated on the upper right. The red, black and blue circles in (A) indicate the positions of voltage pulses from the STM tip. In total, 119 molecules were dissociated: 33 with pulses on the left Br shoulder (red), 58 on the central lobe (black), and 28 on the right Br shoulder (blue). The two STM images in B and C are obtained from two distinct dissociation experiments and show the two possible outcomes, i.e. left or right C–Br bond dissociation. The colored arrows and the percentage values show the measured probability of left and right dissociations at each pulse position. The uncertainty on these values is calculated as the binomial error , where *N* is the total number of cases, *p* and *q* are the probability of left and right bond dissociation, respectively. The error is divided by *N* to yield the relative uncertainty, which is 7% for left and center dissociations and 9% for the right dissociations. (D) Height profiles along the intact and the debrominated molecule, their positions are indicated in (A) and (B), respectively.

The dissociation of a single C–Br bond was induced by voltage pulses from the STM tip. After imaging an intact DBTF molecule, the STM tip is placed at a specific position above the molecule (indicated by circles in Figure 2A) and the feedback loop is disengaged to hold the tip–sample distance constant (set point of 300 pA and 0.6 V). Then, a voltage bias of +2.0 V is applied to the sample (the tip is grounded), and the bond dissociation is identified as an abrupt change in the current-vs-time trace that is recorded during the experiment (Figure S5A). As soon as this single jump of current is observed, the bias is set to zero to prevent further reactions or displacements of the molecule. Accordingly, voltage pulses that show multiple jumps in the current trace–indicating more than one event (i.e., dissociation, translation or rotation) – are discarded (Figure S5B).

The products of the bond dissociation, i.e., the isolated Br atom and the bromo-terfluorene (BTF) fragment, are clearly distinguished in the subsequent STM images (Figure 2B and C). The Br atom (dashed circles) has a “sombrero-shaped” appearance, characteristic for Br on Cu(111).^22^ Due to the chemical change caused by the bond dissociation, the debrominated molecule has a slightly different appearance than the intact precursor. The change in shape is clearly visible in height profiles (Figure 2D) as the Br shoulder of the intact molecule is missing after the voltage pulse. Instead, a depression appears, which is probably an electronic feature due to the molecule–substrate interaction.^9,23,24^ Moreover, the dimethyl lobe next to the debrominated side (see arrow in Figure 2D) has a reduced apparent height after dissociation, indicating a stronger interaction of the dehalogenated side of the molecule with the silver surface. Indeed, the BTF fragment is probably left with an unpaired electron on the carbon atom, and by interacting with the metal substrate it constitutes a surface-stabilized radical,^15,25−30^ which is pinned to the metal surface. The bond between this end of the BTF fragment and the surface is verified by lateral manipulation experiments, which show rotation around a fixed pivot point (Figure S6), but no translation (compare with Figure S2).

To explore the dissociation dynamics, we repeated the experiment many times, each time with a new intact molecule. It is important to note that voltage pulses which caused multiple jumps in the current trace or did not result in any dissociation were excluded. The dissociations are then grouped into three cases, which differ in the tip position with respect to the molecule: the left bromine shoulder (33 cases), the central lobe (58 cases), and the right bromine shoulder (28 cases), indicated in Figure 2A by red, black, and blue circles, respectively. Note that pulses on the left and right Br shoulder are not equivalent. Specifically, there is a mismatch angle between the long axis of the molecule and the [12̅1] symmetry direction of the Ag(111) surface and the two molecular termini at either end have slightly different local surroundings, due to the underlying atomic lattice (Figure 1D, Figures S4 and S16).

Voltage pulses from an STM tip typically affect a molecule only locally, due to the spatial confinement of the tunneling electrons.^6−8^ Accordingly, one might expect that only intramolecular bonds located directly underneath the tip can be dissociated while the rest of the molecule remains unperturbed. In strong contrast to this expectation, we observe that for any of the three pulse positions either the left or the right C–Br bond can dissociate–even the C–Br bond that is most distant from the pulse position. The probabilities of left and right bond dissociation are indicated by arrows in the center of Figure 2. When the tip is placed above one of the two Br shoulders, the probability is highest to break the C–Br bond directly underneath (79% or 71% for pulsing on the left or right shoulder, respectively). However, dissociation of the C–Br bond at the opposite side of the molecule also occurs with remarkable probabilities of 21% or 29%, despite the relatively large distance of 2.9 nm from the pulse position. Additionally, pulses at the center of the molecule can cause both left or right Br dissociation, with equal 50% probability, despite the unequal positions of the two C–Br bonds with respect to the substrate. Therefore, we conclude that the precise location of the Br atoms with respect to the Ag(111) lattice does not affect the dissociation probability of the C–Br bond (see Figure S7 for a discussion about the yields of these processes). This is in contrast to the corrugated Cu(110)^31^ and Au(10,7,7)^32^ surfaces, which were found to lead to selective dissociation, depending on the position of the carbon–halogen bond with respect to the surface.

### Excitation Process

To uncover the mechanism underlying the dissociation of the remote bond from the pulse position, we applied equivalent voltage pulses at the same distance from the C–Br bond (2.9 nm), but on the bare silver surface around the molecule. Despite the equidistant STM tip positioning, these pulses did not trigger any Br dissociation. Electric field and surface-mediated excitations, which could explain remote tip-induced bond dissociations,^9,33,34^ can thus be excluded here. Clearly, the excitation propagates *through* the molecule, from the position where the tunneling electrons are injected to the position where the bond breaks.

**Figure 3:**
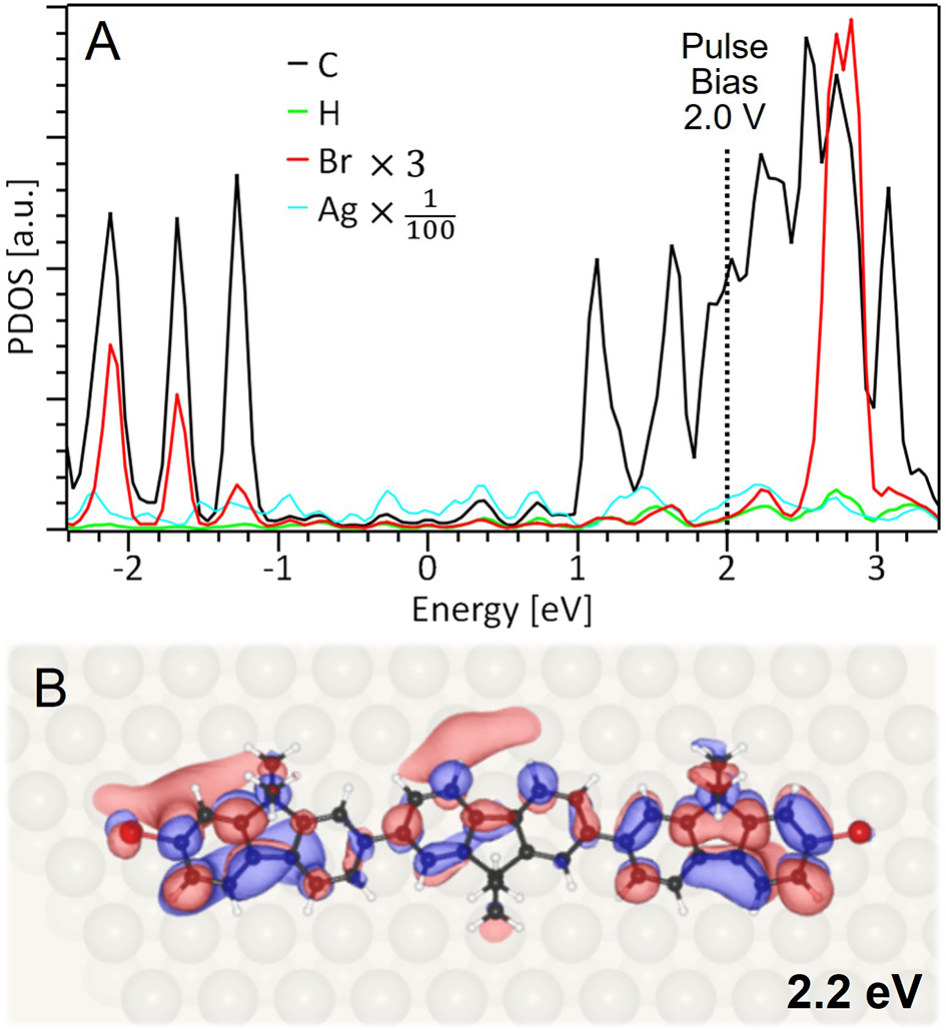
**Calculation of the electronic structure of a DBTF molecule on Ag(111).** (A) Projected density of states (PDOS) of DBTF molecule adsorbed on the Ag(111) surface. The contributions from different atomic species (C, black; H, green; Br, red) and of the silver surface (Ag, blue) are shown separately. The position of the Fermi energy is set to 0 eV. The vertical dotted line indicates the pulse voltage used in the experiments. (B) The projected electron density (isosurface: 0.015 e^–^/Å^3^) of the π* state at 2.2 eV energy (see Figure S9). At this energy the Br contribution shows a peak in the PDOS, which is close to the bias used for the voltage pulses.

One way to induce single bond dissociation with an STM tip is the excitation of molecular vibrations via inelastic electron tunneling.^6^ Such an excitation pathway is observed symmetrically for positive and negative bias polarity.^11^ However, in our case C–Br dissociation can only be induced by positive voltage pulses (applied to the sample while the tip is grounded). Additionally, the threshold voltage for dissociation (1.85 V, see Figure S8) is much higher than the energy of the C–Br stretch vibration (133 meV for bromobenzene adsorbed on Cu(111)^35^) – and in fact than any vibrational energy of carbon–halogen bonds. We therefore relate the C–Br bond dissociation to resonant electron tunneling into an unoccupied electronic state,^28,36^ as supported by calculations (Figure 3 and Figure S9). The voltage of 2.0 V used to induce bond dissociation matches approximately a maximum of the Br contribution to the calculated density of states at 2.2 eV (red curve in Figure 3A). Importantly, the projected electron density at this energy is not only localized on the Br atoms but is instead delocalized over the entire molecule (Figure 3B). This can explain why the tunneling electrons can dissociate the Br atom remote from the injection position, in addition to the one directly underneath the STM tip.

It has been shown in prior studies that electronic states of σ* character are the ones responsible for electron-induced bond dissociation.^14,27,37^ When comparing the calculated density of states for the surface (Figure 3) with the gas phase (Figure S9), orbitals with π* character are found at energies of around 2.0 eV above the Fermi level (i.e., the energy of electron injection), while states with C–Br σ* character are instead present at higher energies of 2.7 eV (see Figure S9). This indicates that the injected electron might first populate a nondissociative π* state and subsequently undergo interconversion to a state with σ* character that is responsible for the C–Br bond dissociation, as reported previously.^36,38,39^ Specifically, π* → σ* interconversion can be facilitated as a result of bond length changes in the nondissociative π* state, bringing both π* and σ* states close in energy, as observed for halobenzene dissociation adsorbed on ice.^40^

Our experiments reveal a larger dissociation probability for the Br underneath the tip as compared to the remote Br at the other end of the molecule (Figure 2). Apparently, the injected electron, once it is in the delocalized π* state (plotted in Figure 3B), does not result in equal probabilities of dissociation of both terminal C–Br bonds. We propose two reasons for this behavior: (1) Different time scales of interconversion and delocalization. If the π* → σ* interconversion occurs faster than delocalization, i.e., before the electron reaches the remote Br, and (2) electrons that are delocalized in the π* state of the planar molecule can dissipate via tunneling to the metal surface before they induce bond dissociation of the remote Br. In both scenarios, the electron has a larger probability to dissociate the bond close to the injection position.

### Dissociation Dynamics

We determined the position and orientation of the intact molecule, with respect to the substrate lattice, from the STM image before dissociation. This information is needed to align all dissociation experiments in the same reference frame. Then, the positions of the two products, i.e., bromine atom and BTF molecular fragment, and orientation of the BTF fragment, were determined from an image of the very same surface area after dissociation. The position of DBTF or BTF is defined by the maximum of the central lobe and their orientation as the angle between the line passing through the two terminal lobes and the horizontal direction. These data were collected for each dissociation experiment (see Figure S10 for a detailed description).

**Figure 4:**
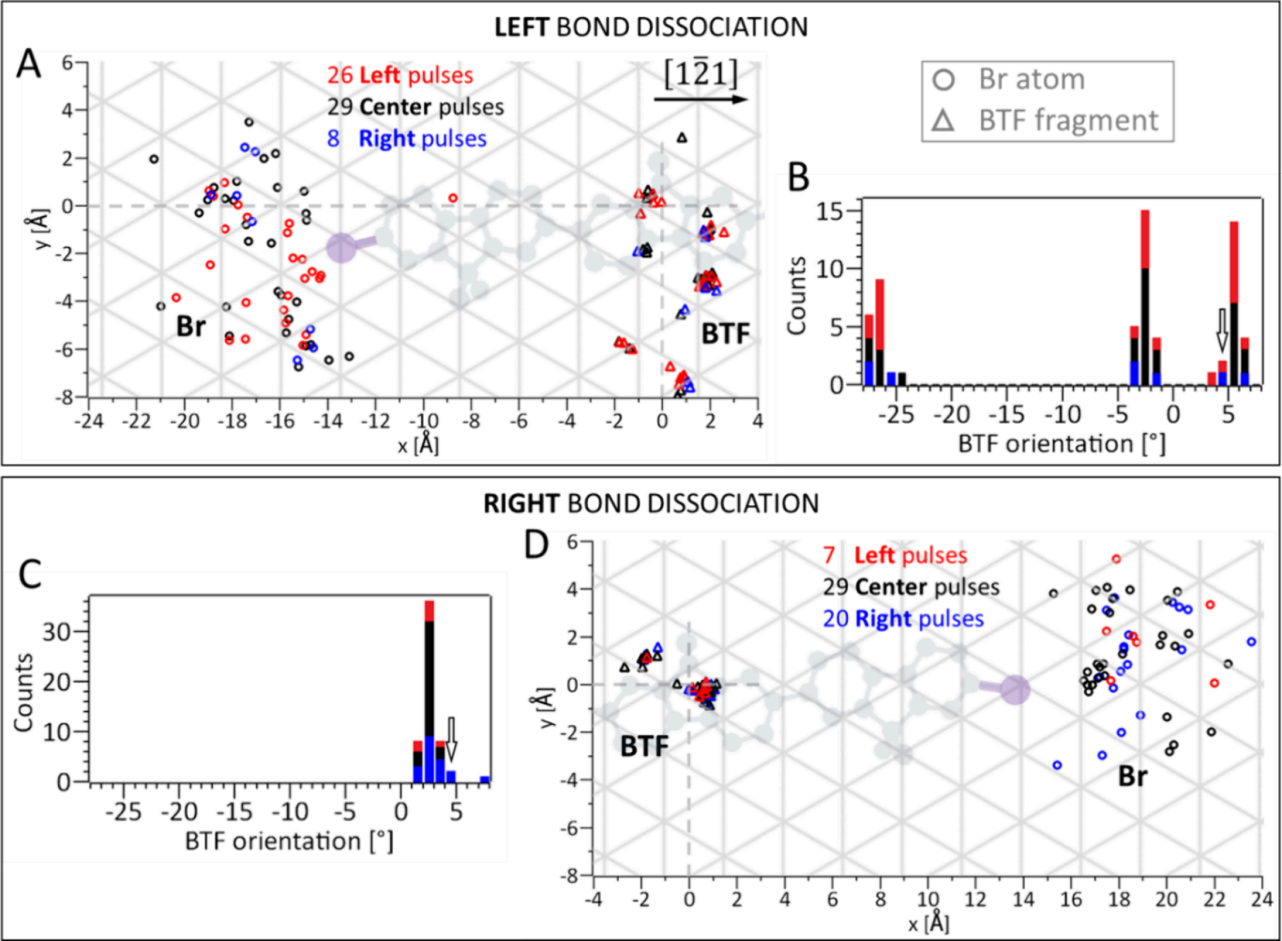
**Position and angle distribution of the fragments.** (A,D) Position distribution of the Br atom and BTF molecular fragment, and (B,C) histograms of the BTF orientation, for the left and right bond dissociation. The data are folded considering the molecule and Ag(111) surface symmetry (Figure S3 and S4). In A and D, the position of the center of the DBTF molecule before bond dissociation defines the (0,0) origin of the coordinate system. The chemical structure of the intact molecule is drawn in gray (with the Br atoms in purple) and the Ag(111) is plotted as a gray lattice, where each crossing point represents a silver atom. The alignment between STM image of the molecule, molecular structure and Ag(111) is described in Figure S4. Position of Br atoms and of BTF central lobe are indicated by circles and triangles, respectively. The three different colors are used to differentiate the data based on the position of the pulse (as in Figure 2A). In the histograms B and C, the columns for the three cases are added on top of each other, the [12̅1] direction is at 0°, and the arrow indicates the orientation of the intact molecule.

The spatial distribution of the products on the surface is plotted in Figure 4A and D for the left and right bond dissociations, respectively. In addition, the final orientations of the BTF fragment are depicted in Figure 4B and C. Analysis of the scatter plots and histograms of Figure 4A-D for the three pulse positions (labeled **left**, **center**, and **right** with different colors) shows that the final positions of the two products follow a similar distribution regardless of the pulse position. Hence, the dissociation dynamics are independent of the position of electron injection into the molecule.

While the pulse position does not play a role, the slightly different alignment of the left and right molecular termini with respect to the Ag(111) lattice (Figure 1D) creates an unequal chemical surrounding, causing different rotations and displacements (upper vs lower panel in Figure 4). For the left bond dissociation, the Br atoms are distributed exclusively on the left side of the original C–Br bond, while for the right dissociation they are only on the right side (Figure 4A and D, respectively, with the C–Br bonds indicated in violet), located at an average distance of about 5 Å from their original position in both cases (Figure S11A). It has been demonstrated that the distribution of halogen atoms, dissociated from a halobenzene on Cu(110), is strongly correlated with the direction of the broken carbon–halogen bond, even for different orientations of the molecule on this corrugated surface.^41^ Our results demonstrate a similar correlation between Br distribution and the prior C–Br bond orientation. However, the Br distribution exhibits a broad spread in angles (about ±60° for the left dissociation and ±30° for the right dissociation, see Figure S11B). To understand this, we looked for a correlation between Br positions and orientations of the BTF fragment, i.e. does the Br atom preferably move along the BTF orientation? We found that the positions of Br atoms are widely distributed across different angles, regardless of the BTF orientation (Figure S12). Thus, no such correlation is present, suggesting that Br atoms are randomly deflected by interaction with silver atoms of the flat Ag(111) surface along no preferential direction, in agreement with previous work.^37,41^

In contrast to the Br atoms, the position distribution of the BTF fragment groups into defined clusters with six major ones for the left and two for the right dissociation (Figure 4A and D). The BTF fragment is displaced by about 1 and 2 Å for the right bond dissociation, while for the left bond dissociation larger distances between about 1 and 8 Å were observed. However, the larger displacements do not correspond to translations only, but rather to rotations of the molecular fragment (as discussed in Figure S12). By inspecting the histogram of the BTF orientation angles for the left bond dissociation (Figure 4B) it becomes clear that in 66% of the cases the fragment undergoes substantial rotational movement, reorienting the BTF fragment to −2.5° and −27.0°. Hence, one direction of rotation is preferred (details in Figure S13), similar to what has been observed for 1,3-diiodobenzene on Cu(110).^16^ In the other 34% of the left dissociations, the BTF orientation remains essentially unchanged. For the right dissociations, no relevant rotations of the fragment were observed at all (Figure 4C).

**Figure 5:**
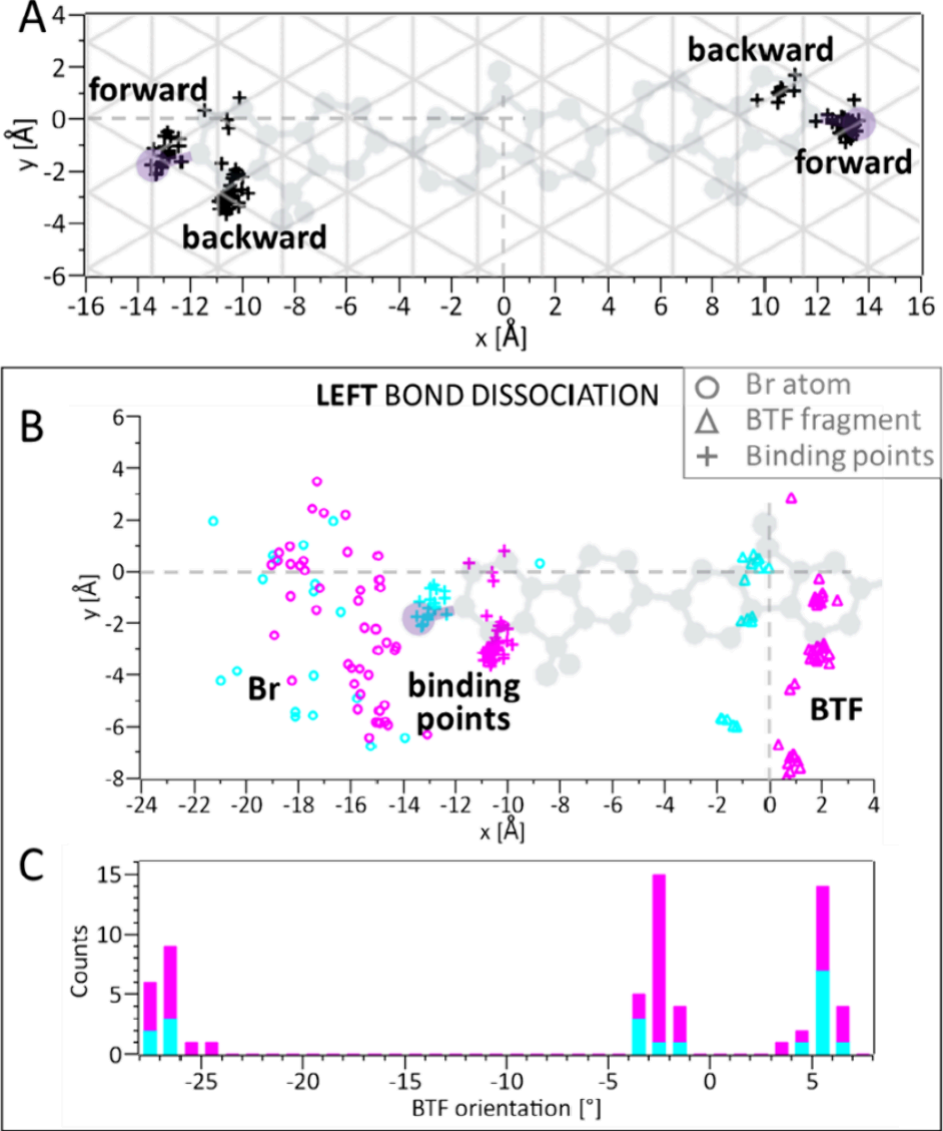
**Color coding according to BTF binding point.** (A) Scatter plot of the binding point position obtained for each dissociation experiment (details in Figure S14). For both left and right dissociation, there are clusters of binding positions, indicated with “forward” and “backward”. The molecular structure of the intact molecule and the substrate lattice are plotted in the background. (B–C) The same scattering plot and histogram as in Figure 4A-B, but color-coded to the binding points of the BTF fragment (results for right dissociations in Figure S15): cyan for the cluster defined as “forward”, and magenta for “backward”. Circles, triangles and crosses in (B) represent the position of the bromine, molecular fragment, and binding point, respectively. The intact molecular structure is shown in gray. (C) Orientation of the molecular fragment after dissociation. The columns for the two cases are added on top of each other.

The BTF rotates around a fixed pivot point (Figure S6), which can be determined for each dissociation experiment (details in Figure S14) and is plotted in Figure 5A. For both left and right dissociations, the distribution of pivot points is divided into two major clusters, localized around positions of surface silver atoms, suggesting that after dissociation the debrominated side of the molecule binds to one of the nearest silver atoms. This interaction might be caused by the metal surface electrons that readily couple to the free valence of the debrominated side of the molecule.^23−25^ As a consequence, the BTF fragment becomes a surface-stabilized radical with a binding position defined by the silver lattice.

Additionally, we color-coded the Br and BTF position distribution and the BTF orientation histograms (given in Figure 4) according to the binding points identified in Figure 5A. The data are colored in cyan and magenta for the forward and backward binding point, respectively. We added also to the plot the distribution of pivot points (crosses). The resulting plot and histogram for the left bond dissociations are shown in Figure 5B–C (Figure S15 for the right bond dissociations). The BTF positions (triangles) separate into two sets, corresponding to the binding point. The BTF final orientations (histogram) are independent from the binding point as they show the same angles (Figure 5C). The position of Br atoms (circles) is also not related to the binding point of BTF fragments. This observation adds to the finding that the position of the Br atom shows no correlation with the orientation of the molecular fragment (Figure S12). Therefore, we conclude that immediately after bond dissociation the two products follow uncorrelated dynamics.

Finally, we considered momentum conservation, as discussed for gas phase dissociation experiments.^1,42,43^ According to this principle, it could be expected that the BTF fragment moves always in opposite direction to the Br atom as observed in the gas phase.^44^ However, this is not always the case as the binding positions indicate not only a “backward”, but also a “forward” – i.e. toward the Br atom–motion of the BTF fragment (Figure 5A). The “forward” cases are not rare but amount to 30% and 79% for the left and right bond dissociation, respectively. Hence, the BTF can move, counterintuitively, in the same direction as the Br atom, to reach one of the nearest Ag atoms, i.e. possible binding positions, demonstrating that the molecule–surface interaction strongly affects the dissociation dynamics on metal surfaces.

## Conclusions

Based on our observations, we conclude that the excitation responsible for C–Br dissociation in DBTF molecules can be mediated by a delocalized molecular orbital, resulting in the cleavage of a rather distant C–Br bond, about 3 nm away from the pulse position. This finding contributes to the fundamental understanding of how a molecular excitation event guides the subsequent dissociation of a chemical bond. Our experiments clearly show that, right after C–Br bond breaking, the dynamics of the Br atom and the BTF fragment are uncorrelated. The Br atom is scattered, due to the interaction with the surface atoms that deflect its trajectory, while the BTF fragment binds its debrominated side to one of the nearest silver surface atoms, which acts as pivot point, and can dissipate its energy through rotation. Furthermore, the dynamics of the dissociation do not depend on the position of the electron injection into the molecule. Our results provide a complete description of the dissociation dynamics of single bonds on a metal surface, elucidating the important role of the metal support. Understanding the dynamics of dissociation products on a surface could be employed to precisely localize chemical reactions and to orchestrate reaction cascades, paving the way toward engineering of multicomponent molecular architectures.

## Methods

The (111) surface of a silver single-crystal was prepared by several cycles of argon ion sputtering and subsequent annealing to obtain large, clean and atomically flat terraces. DBTF (dibromo-terfluorene) molecules were deposited by evaporation from a Knudsen cell under ultrahigh vacuum onto the Ag(111) surface held at room temperature. Then, the sample was cooled and transferred to the STM stage where imaging and manipulation were done at 7 K.

Routine STM tip forming was done to improve the tip apex quality by soft indentation into metallic surface areas, leading to a sharp, metallic tip. Note that this procedure did not change the results of the manipulation experiments in this work. The dissociation of a single C–Br bond was induced by voltage pulses from the STM tip. After imaging an intact DBTF molecule, the STM tip is placed at the desired lateral position (indicated by circles in Figure 2A) above the molecule at a height that is determined by the set point (300 pA and 0.6 V). The STM feedback loop is then disengaged to keep the tip–sample distance constant and a bias voltage of +2.0 V is applied to the sample (while the tip is grounded). The change in applied bias voltage +0.6 V to +2.0 V and vice versa happens within about 2 ms. A bond dissociation is identified as an abrupt change in the current-vs-time trace that is recorded during the experiment (Figure S5A). As soon as this single jump of current is observed, the bias is set to zero to prevent further reactions or displacements of the molecule. Accordingly, voltage pulses that show multiple jumps in the current trace–indicating more than one event (i.e., dissociation, translation or rotation) – are discarded (Figure S5B).

Position and orientation of the intact molecule with regard to the substrate lattice were identified from the STM image before dissociation. This information is needed to align all dissociation experiments in the same reference frame. Then, the positions of the two products, i.e., bromine atom and BTF molecular fragment, and orientation of the BTF fragment, were determined from an image of the very same surface area after dissociation. The position of DBTF or BTF is defined by the maximum of the central lobe, their orientation as the angle between the line passing through the two terminal lobes and the horizontal direction. These data were collected for each dissociation experiment (see Figure S10 for a detailed description).

## Supporting Information Available

Experimental procedures including self-assembled molecular islands, lateral manipulation of single DBTF molecules, adsorption orientation and configuration of single DBTF molecules, current-vs-time trace during a voltage pulse, lateral manipulation of a DTF molecular fragment after Br dissociation, yield of dissociation, threshold voltage for dissociation, calculation of the electronic structure of a DBTF molecule in the gas phase, position and orientation measurement before and after dissociation, distribution of scattering angle and distance of the bromine atoms, color coding according to BTF orientation, rotations of the debrominated molecular fragment, determining the pivot point of a debrominated molecule, and color coding according to BTF binding point (PDF)

## Notes

A preprint version of a very similar version of this manuscript is available online.^45^
