# Supplementary material for: Bond Dissociation Dynamics of Single Molecules on a Metal Surface
Source: ACS Nano. 2025 Mar 5;19(10):10255–62. doi: 10.1021/acsnano.4c17652 (PMC11924323; doi:10.1021/acsnano.4c17652)
Supplement: Supplementary file 1 — nn4c17652_si_001.pdf [file nn4c17652_si_001.pdf]

Bond dissociation dynamics of single molecules on a metal surface

Donato Civita[1], Matthew Timm[1], Jutta Schwarz[2], Stefan Hecht[2], and Leonhard Grill[1*]

[1]*Department of Physical Chemistry, University of Graz, Heinrichstraße 28, 8010 Graz, Austria*

[2]*Department of Chemistry & Center for the Science of Materials Berlin, Humboldt University Berlin, Brook-Taylor-Str. 2, 12489 Berlin, Germany*

Contents

1. Self-assembled molecular islands S2
2. Lateral manipulation of single DBTF molecules S3
3. Adsorption orientation of single DBTF molecules S4
4. Adsorption configuration of single DBTF molecules S6
5. Current-vs-time trace during a voltage pulse S7
6. Lateral manipulation of a DTF molecular fragment after Br dissociation S8
7. Yield of dissociation S9
8. Threshold voltage for dissociation S11
9. Calculation of the electronic structure of a DBTF molecule in the gas phase S12
10. Position and orientation measurement before and after dissociation S13
11. Distribution of scattering angle and distance of the bromine atoms S14
12. Colour coding according to BTF orientation S15
13. Rotations of the de-brominated molecular fragment S16
14. Determining the pivot point of a de-brominated molecule S17
15. Colour coding according to BTF binding point S18
16. Calculated adsorption geometry for DBTF S19

References S20

1. Self-assembled molecular islands

DBTF molecules are evaporated onto the clean Ag(111) substrate held at room temperature. Subsequent STM imaging at 7K shows self-assembled molecular islands, typically of elongated shape, as shown in Fig. S1.

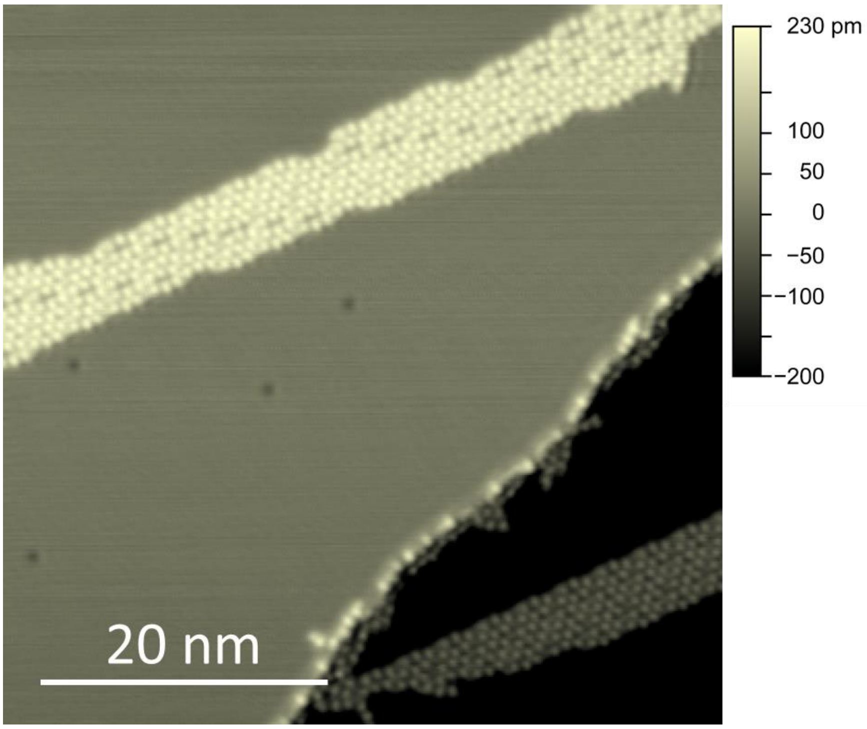

Figure S1. *STM image of the Ag(111) sample with adsorbed DBTF molecules (0.6 V, 300 pA). A large terrace occupies most of the image, and a diagonal step edge, decorated with DBTF molecules, separates another terrace. Two elongated islands of DBTF molecules are present on the two terraces.*

2. Lateral manipulation of single DBTF molecules

A single DBTF molecule can be extracted from a molecular island by lateral manipulation with the STM tip as shown in Fig. S2. Lateral manipulation is executed at constant current of 80 nA, with 0.1 V voltage bias, and 40 Å/s lateral speed. If the tip height, which is recorded during manipulation, shows a characteristic sawtooth shape then the molecule was successfully detached from the island and dragged to the free terrace. A subsequent STM image (Fig. S2B) then confirms the successful manipulation, as a single molecule appears on the terrace.

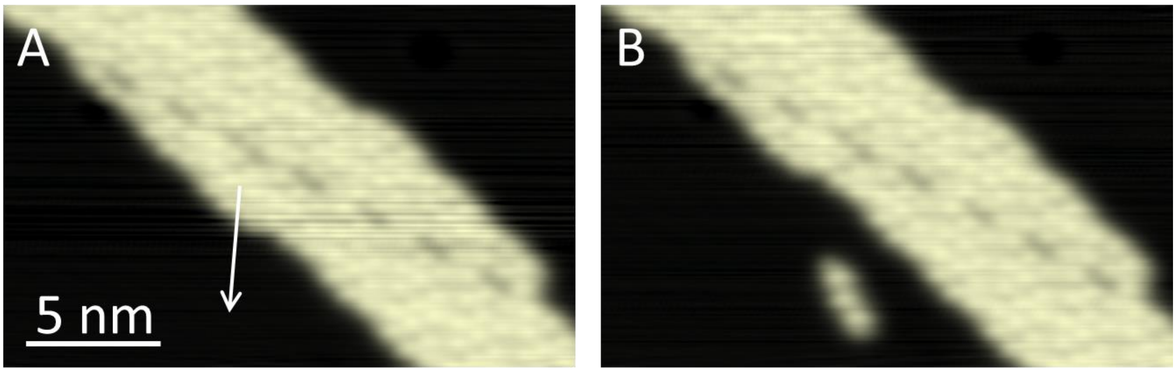

Figure S2. *Two STM images (0.6 V, 300 pA) of the same surface area before (A) and after (B) a lateral manipulation with the STM tip. An elongated island of DBTF molecules is visible in the scanned area. The path followed by the STM tip during the lateral manipulation is indicated with a white arrow in A. The single molecule detached from the island is visible in B.*

3. Adsorption orientation of single DBTF molecules

To obtain statistical information on the stable molecular orientations on the surface, we repeatedly manipulated 54 individual DBTF molecules on free clean terraces of the Ag(111) surface to reorient each of them several times. For each molecule, the orientation was determined after each manipulation, rendering 199 measurements in total. The orientation is defined as the angle between the horizontal direction in the images, which is the $[1\bar{2}1]$ direction of the substrate (Fig. S3B), and the line passing through the two side lobes of the molecule (see Fig. S10). This direction is defined in our analysis as 0° and counter-clockwise rotations correspond to positive changes in the angle.

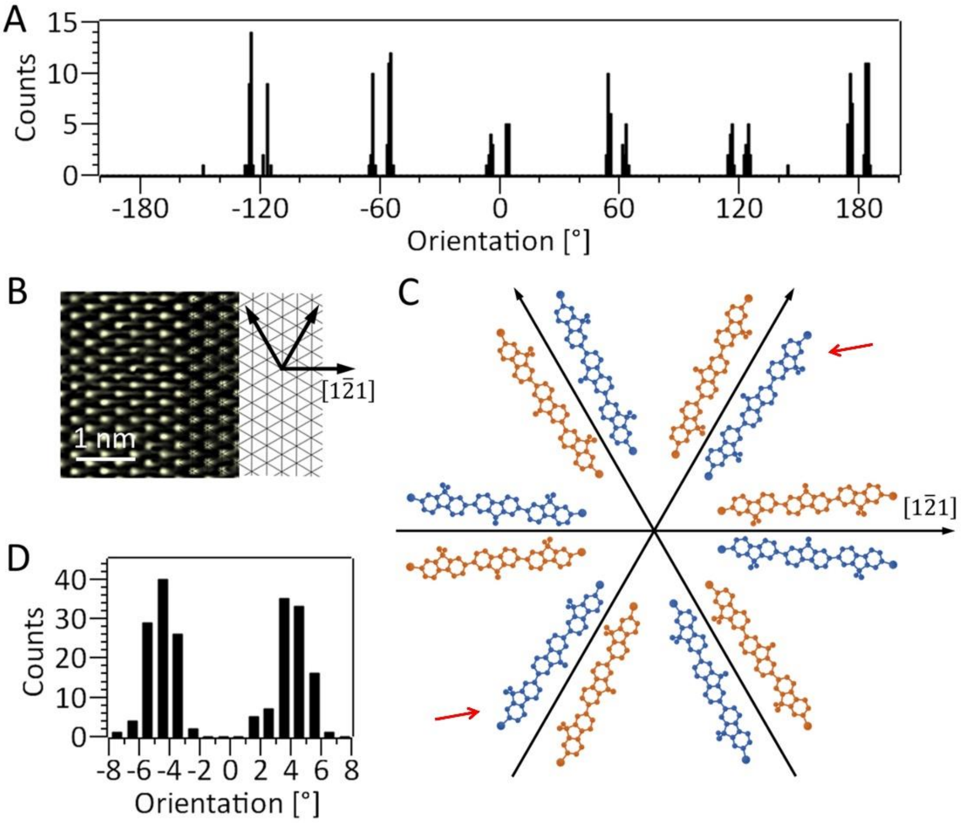

Figure S3. *(A) Histogram of the measured orientation angle of the single intact DBTF molecule on the Ag(111) surface. (B) Atomic resolution STM image of the Ag(111) substrate, which is rotated to have the* $[1\bar{2}1]$ *direction horizontal. From this image the high symmetry directions of the surface can be obtained. (C) Scheme of the twelve adsorption orientations. Equivalent configurations have the same colour, i.e., orange or blue. (D) Folded data, considering the 6-fold symmetry of the Ag(111). In this way all peaks collapse in only two maxima, which indicate the two possible orientations of the single DBTF molecules with respect to the* $\langle 121 \rangle$ *high symmetry directions of the Ag(111) surface.*

The histogram in Fig. S3A shows that the orientation values group in two peaks close to the $\langle 121 \rangle$ high symmetry directions of the substrate. These directions are at 0°, ±60°, ±120° and +180° as indicated in Fig. S3B where they are extracted from the atomic resolution image of the Ag(111) surface. All molecules end up in one of these orientations after being manipulated

on the surface and no other adsorption orientations were found. Hence, these are the stable orientations, which are shown schematically in Fig. S3C. Notice that mirroring the molecule along its long axis is equivalent to a 180° rotation of the molecule (an example is marked by arrows in Fig. S3C), demonstrating that all possible configurations are considered. In addition, a metastable orientation was observed but the molecules in this orientation are highly mobile, as reported previously.[1]

Considering the 6-fold symmetry of the Ag(111) surface, all measured data can be folded into the histogram in Fig. S3D. In this way the peaks collapse into only two peaks, which indicate two possible chiral orientations of the 'single' DBTF molecules on the Ag(111) surface, at ±4.5° (±1°) with respect to the $[1\bar{2}1]$ direction. The data are further folded considering the mirror symmetry of the molecule (details in Fig. S4) to obtain the histogram shown in Fig. 1C of the main text.

4. Adsorption configuration of single DBTF molecules

To obtain the precise adsorption configuration, six Br atoms were positioned around a DBTF molecule on a free terrace of the Ag(111) surface (Fig. S4A). The correctly scaled molecular structure has been superimposed and aligned with the image features. The lattice of the surface atoms is overlaid to have all the Br atoms in a 3-fold fcc or hcp site of the surface (note that there is only one possible lattice position/orientation in which all Br atoms are placed in fcc and hcp positions), which is the known adsorption position of atomic Br on Ag(111).[2] This analysis reveals the adsorption position of the molecule with respect to the surface lattice. The adsorption configuration found in this way is presented in B as the orange molecular structure. This adsorption configuration corresponds to the peak at +4.5° in the distribution of molecule orientations in Fig. S3C. We can observe that the left and right C-Br bonds of the molecule have different alignments with the underlying silver lattice.

From other STM images, similar to Fig. S4A, the second adsorption configuration is found and represented in Fig. S4B by the blue molecular structure. This second adsorption configuration corresponds to the peak at -4.5° in the distribution of molecule orientations in Fig. S3C. It is possible to show that these two adsorption configurations are equivalent when mirroring along the vertical axes (blue dashed line), due to the molecular symmetry. Indeed, the orange adsorption configuration can be obtained by mirroring the blue one. Therefore, we reflected all STM images of the blue configuration to fold all data in a unique adsorption configuration (corresponding to the orange configuration). In this way, from Fig. S3C we obtain the histogram in Fig. 1C (main text), and all subsequent analysis can be done considering a unique adsorption configuration (orange), only distinguishing between the left and right C-Br bond.

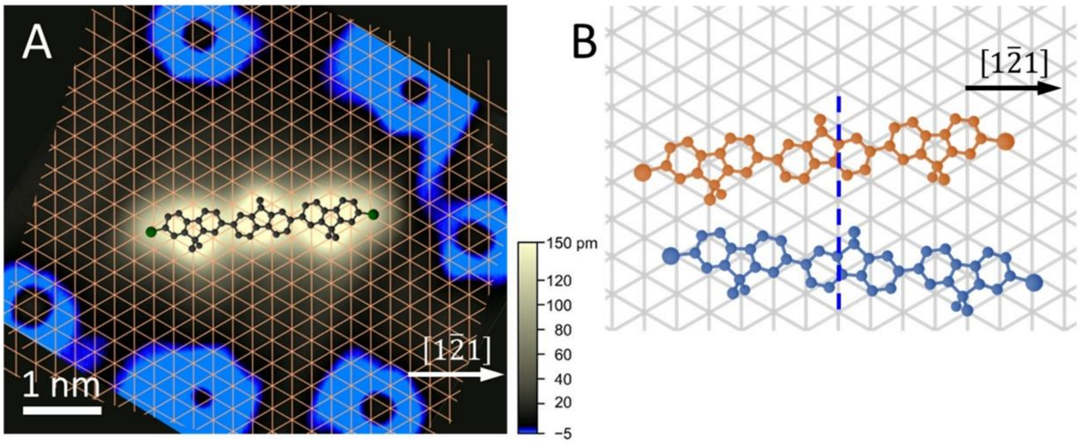

Figure S4. *(A) STM image of a DBTF molecule surrounded by six bromine atoms (0.2 V, 1.0 nA). The Br atoms are visible as circular depressions in blue. The image is rotated to have the* $[1\bar{2}1]$ *direction horizontal. The molecular structure of DBTF is superimposed over the image (black and green circles represent carbon and bromine atoms, respectively). The Ag(111) surface lattice is also overlaid, where the cross points indicate the position of silver atoms. (B) The two adsorption configurations at +4.5° (in orange) and at -4.5° (in blue).*

5. Current-vs-time trace during a voltage pulse

During the voltage pulses (2.0 V is applied to the sample) at constant tip height, the tunnelling current is recorded in time while the vertical and lateral positions of the tip are fixed above the molecule. With these parameters, the rate of dissociation is relatively slow so that bond dissociation occurs typically within the first 20 seconds. In Fig. S5A a typical I(t) curve is shown, revealing a sudden current drop at 11.55 s, which indicates a C-Br bond dissociation. This is confirmed by a subsequent image of the same area, which shows the two distinct fragments of the dissociation. In all cases, as soon as an abrupt change in the current trace is observed, the voltage pulse is stopped. In this way only one molecular event takes place, as multiple events, like rotation or translation of the molecule, would result in additional jumps in the current trace. Experiments with multiple current jumps (an example is given in Fig. S5B) are discarded from our data.

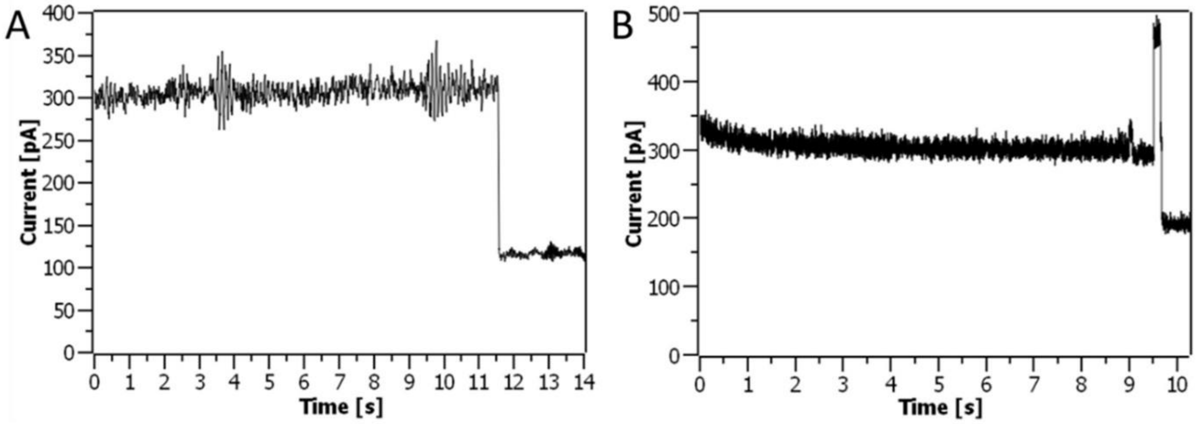

Figure S5. *Current-vs-time traces during a voltage pulse used for the electron-induced single bond dissociation. (A) shows an example with a single jump in the current, while in (B) an example with multiple jumps is plotted.*

6. Lateral manipulation of a DTF molecular fragment after Br dissociation

After C-Br bond dissociation, the de-brominated molecule can be laterally manipulated with the STM tip. A sequence is shown in Fig. S6 where an STM image is recorded after each manipulation step. The de-brominated terminus of the molecule has a lower apparent height (see Fig. 2D), and on that side a depression is visible (blue in Fig. S6). It can be clearly seen that the de-brominated DBTF molecule rotates around a fixed pivot point (note that Br atom at the bottom of the image that acts as fixed reference point).

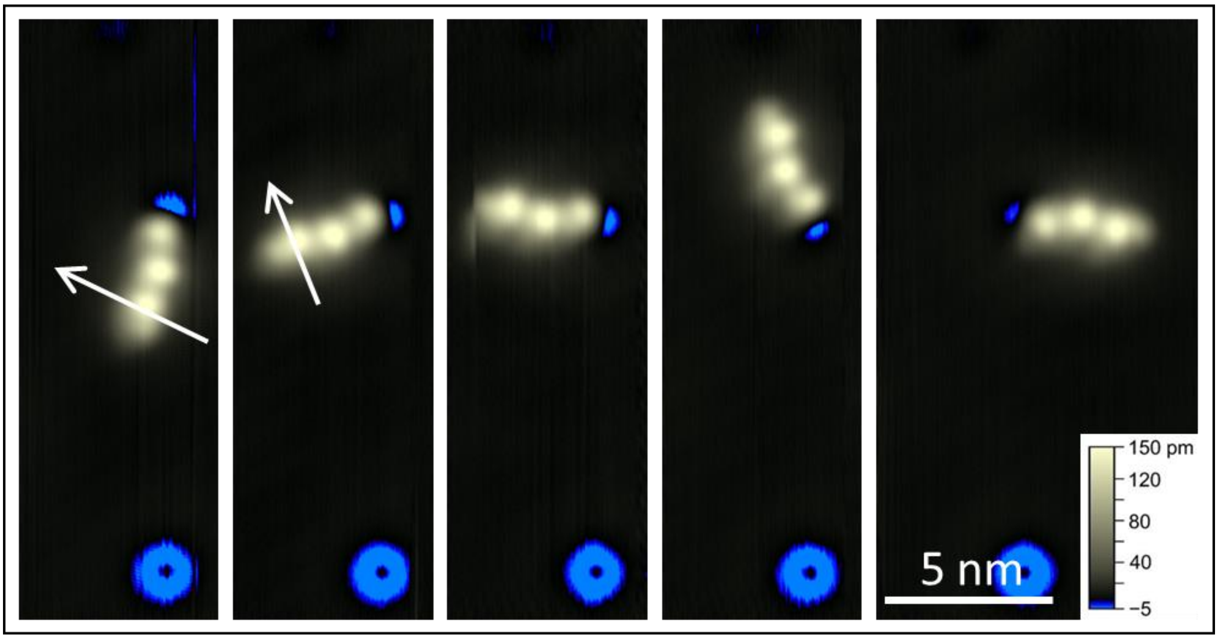

Figure S6. *Series of subsequent STM images (0.2 V, 500 pA), each recorded after a lateral STM manipulation (0.1 V, 80 nA) of a de-brominated DBTF molecule. The molecular fragment is recognizable from the three bright lobes with a blue depression on one side. A single Br atom in the bottom of the images appears as blue "sombrero-shaped" depression that remains at the same position, acting as fixed reference point. The white arrow in the first two images indicates the path of the tip during the lateral manipulation.*

7. Yield of dissociation

The following analysis is based on the statistical model introduced by Ho[3], then detailed by Riedel[4], and implemented by Polanyi[5] to describe electron-induced processes with two possible outcomes, *i.e.* a "double reaction pathway". This is the case for electron-induced C-Br bond dissociation in DBTF molecules on Ag(111). In this system, the two possible outcomes of a voltage pulse are dissociation of the "left" or the "right" Br substituent (see scheme in Fig. S7A). The voltage pulse can be applied at two different positions of the molecule, which are the central fluorene lobe or the Br atom ("centre" and "side" in the scheme). For this analysis we will not distinguish between pulses on the left or the right Br shoulder, because the different alignments of the two termini of the molecule with respect to the surface lattice do not influence the dissociation probability of one or the other bond. This is shown from (1) pulsing experiments on the centre of the molecule give 50% probability of dissociation of either side (see Fig. 2 in the main text) and from (2) the observation that the dynamics of dissociation is independent on the pulse position (see Fig. 4 in the main text).

The yield for the two possible outcomes of each dissociation experiment, *i.e.* the probability of reaction per electron, is given by[5]:

$$p_L = \frac{e}{I\tau}\frac{N_L}{N_{TOT}}$$

$$p_R = \frac{e}{I\tau}\frac{N_R}{N_{TOT}}$$

where the two possible outcomes refer to the "left" (L) and "right" (R) bond dissociation. $N_{L/R}$ are the numbers of successful left/right dissociations, while $N_{TOT} = N_L + N_R$. I is the average current before the discontinuity, e is the electron charge and τ is the decay constant of the probability to induce a bond dissociation.

A

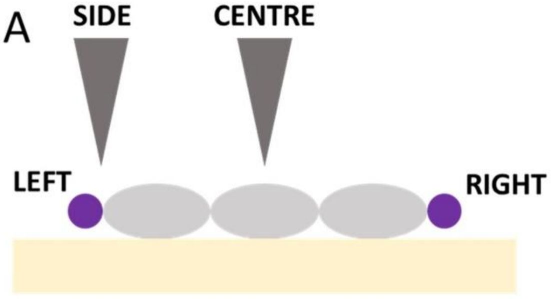

B

$10^{-12}\frac{events}{electron}$	LEFT	RIGHT
CENTRE	1.8 ± 0.8	1.8 ± 0.8
SIDE	7 ± 4	3 ± 2

Figure S7. *(A) Scheme (side view) of a dissociation experiment with the different STM tip positions with respect to the molecule ("side" and "centre"). The two possible C-Br bond dissociations at either end of the molecule are indicated ("left" or "right"). The grey triangles represent the STM tip, the light-grey ovals are the fluorene groups of the DBTF molecule and the violet circles are the Br atoms (the yellow rectangle is the silver substrate). (B) Table showing the dissociation yield (in 10^{-12} events/electron) for the different cases.*

To obtain I and τ, we analyse the electric current trace recorded for each dissociation experiment (an example is shown in Fig. S5A). The mean values of the current before the sudden jump, measured for each pulse, is averaged over all the experiments, and we obtain 5 $\pm$ 1 nA and 3 $\pm$ 1 nA for the pulses on the center and on the side of the molecule, respectively. The times elapsed from the beginning of the pulse to the sudden jump of the current for each dissociation experiments are collected in an histogram. This histogram is fitted with an exponential decay from where the decay constant is extracted. The decay constant obtained for the pulses on the center of the molecule is 9 $\pm$ 2 s and the one for pulses on the side is 6 $\pm$ 1 s, allowing calculation of the yield. The resulting values, presented in Fig. S7B, show that pulses on the centre of the molecule give same yield for “left” or “right” dissociation. However, we find a clearly higher yield (about doubled) for dissociation of the C-Br bond *underneath* the tip (i.e. pulse on the “side” and dissociation of the “left” C-Br bond) as compared to dissociation of the *remote* bond (*i.e.* “right”).

8. Threshold voltage for dissociation

To measure the threshold voltage required to induce a C-Br bond dissociation in DBTF molecules, we use a method that has been reported previously.[6,7] The STM tip is positioned (at constant height) over the target bond of the molecule and the voltage bias applied to the sample is ramped from 0.20 to 2.00 V at a slow rate of 0.01 V/s. The resulting I(V) trace shows a sudden drop around 1.80 V, as in Fig. S8. The subsequent image shows the Br atom and the molecular fragment separated, as in Fig. 2 (main text).

Repeating this test many times, the average threshold bias for C–Br bond breaking is statistically determined as 1.85 ± 0.1 V. However, if the same test is repeated with negative bias voltages, the C-Br dissociation is never observed (for values between 0 V and –3.00 V; note that voltage pulses above ±3.00 V induce decomposition of the molecule). Since the threshold voltage for C-Br dissociation is not symmetric for positive and negative bias polarities, we can exclude inelastic electron tunnelling as an excitation mechanism.[3] Instead, the C-Br bond dissociation in DBTF molecules seems to be mediated by resonant tunnelling into an unoccupied electronic state of the molecule (see discussion about Fig. 3 in the main text for more details).

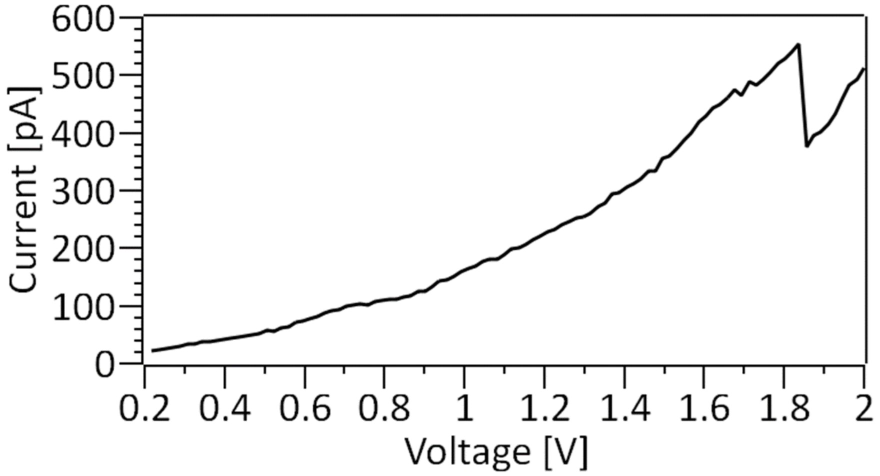

Figure S8. *Example of an I(V) ramp measured on a single DBTF molecule to obtain the threshold voltage bias for dissociation of a carbon-bromine bond.*

9. Calculation of the electronic structure of a DBTF molecule in the gas phase

Density functional theoretical calculation were performed by Qifan Chen and Pavel Jelinek (Czech Academy of Sciences) to optimize DBTF on Ag(111) using the all-electron Fritz-Haber Institute Ab-Initio Materials Simulation (FHI-AIMS) code[8] within XC PBE functional[9] and light basis set. The structural optimization was done using a (7×13) slab model of Ag(111) with a DBTF molecule, where the surface was represented by 3 layers, of which the last Ag layer was fixed during the optimization. In total, the slab model consisted of 356 atoms. Van der Waals (vdW) correction based on Hirshfeld partitioning of the electron density described in Tkatchenko and Scheffler[10] was added to account for dispersion forces. The atomic structures were thoroughly relaxed until the Hellman-Feynman forces were smaller than 10^{-2} eV·Å^{-1}. The relaxed structures were found by exploring different initial conditions and selecting the one with the lowest potential energy. Only the Γ k-point was used for integration in the Brillouin zone.

Calculations of the DBTF molecule in the gas phase show that C-Br σ* orbitals are at higher energies than the π* orbitals (Fig. S9). This is emphasized in Fig. S9B, where dashed black lines over the electron densities at 2.70 eV show a nodal plane between the C and Br atoms, which is characteristic for C-Br σ* orbitals.

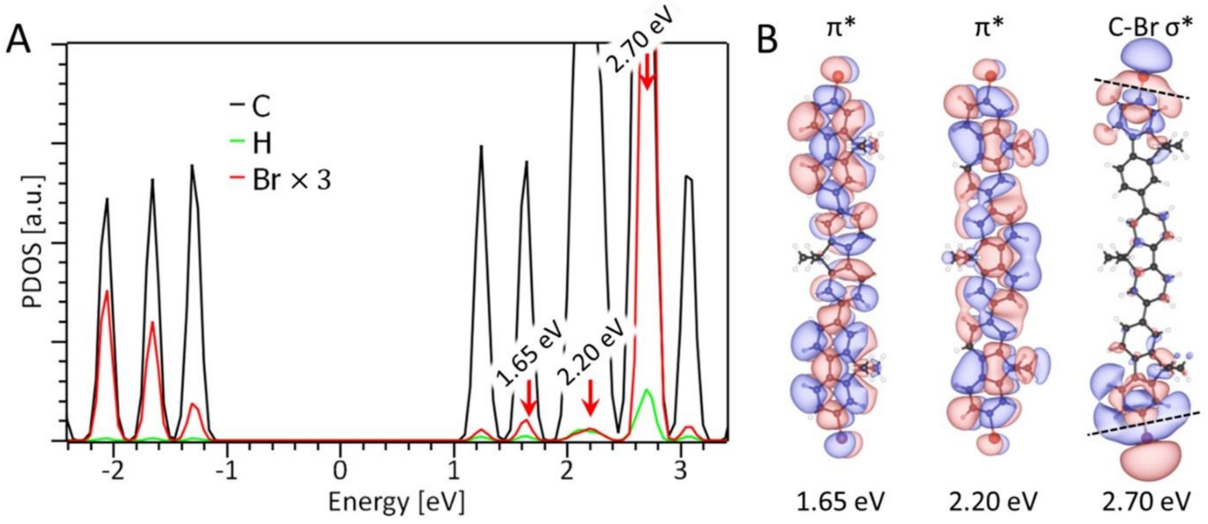

Figure S9. *(A) Contributions of the C atoms (black), Br atoms (red) and H atoms (green) to the projected density of states (PDOS) of gas-phase DBTF. The origin of the horizontal axis is set so that the energy calculated for the HOMO in the gas phase is at the same energy as the calculated HOMO on the surface (i.e., the first peak below the Fermi energy). Unoccupied orbitals with a noticeable Br contribution (and the energy of these states) are indicated in (A) by red arrows; the corresponding projected electron densities at these energies (isosurface: 0.015 e^{-} ·Å^{-3}) are shown in (B).*

10. Position and orientation measurement before and after dissociation

STM imaging before and after the C-Br bond dissociation is necessary to determine the change in the position and orientation of the molecule and the position of the Br atom. In Fig. S10, STM images of the same molecule before and after de-bromination are shown. It is important to note that we ensured that, during a voltage pulse, the molecule does not rotate or move before the dissociation event by discarding experiments with multiple jumps in the current trace (see Fig. S5). The collected data are indicated on the images by coloured dots and white lines. The position of the molecule (intact or de-brominated) is identified through the maximum of the central lobe (blue dot). The orientation is defined as the angle between the horizontal direction and the line passing through the two side lobes (red dots). Positive angles are measured in the counter-clockwise direction. The position of a reference point (green dot in the island at the right) is also recorded to account for possible drift during imaging. These data are collected after the experiments with a semi-automated Python program.

Position and orientation of the intact molecule serve to align all dissociation experiments in the same reference frame. Indeed, all data are rotated and shifted (and eventually mirrored as explained in Fig. S4) to have the $[1\bar{2}1]$ substrate direction horizontal, the intact molecule at +4.5° and with the central lobe at the (0,0) position, which is the common starting condition for all intact molecules.

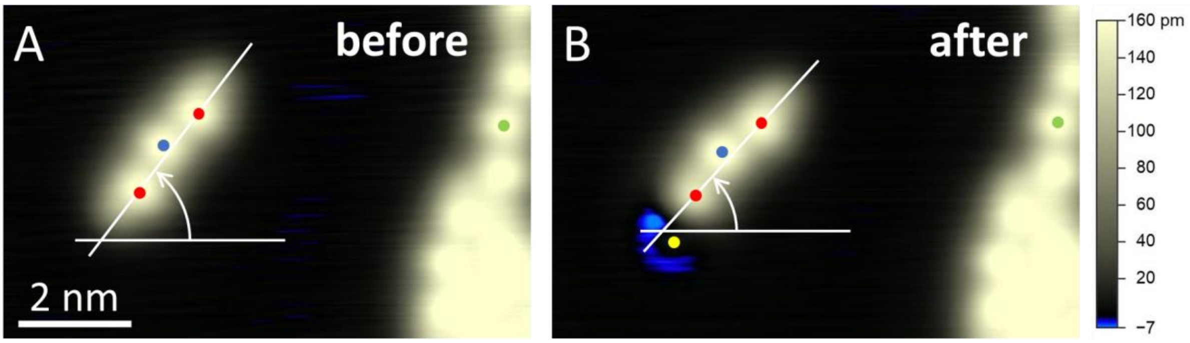

Figure S10. *(A-B) STM images (0.6 V, 300 pA) of a single DBTF molecule before and after, respectively, de-bromination. Other DBTF molecules are visible as reference at the right. The blue dots indicate the position of the central lobe of the molecule (molecular fragment) before (after) the pulse. The red dots indicate the position of the side lobes, used to measure the orientation angle of the molecule (molecular fragment). The orientation angle is indicated with white arrow in the images. The yellow dot in B indicates the position of the Br atom after the dissociation. The green dots in A and B indicate the position of the same reference point (in this case is a lobe of another DBTF molecule).*

11. Distribution of scattering angle and distance of the bromine atoms

The two histograms in Fig. S11 show the distribution of scattering distances and angles of the bromine atoms after C-Br bond dissociation. They are built with the modulus and angles of the vector joining the initial to the final position of the Br atom. The initial position of the Br is the one in the intact molecule, which is measured from the correctly scaled molecular structure (see Br in the molecular structure in Fig. 4A and D of the main text). The final positions are those plotted with circles in Fig. 4A and D on the main text. The two colours of the bars refer to the left (grey) or right (black) bond dissociation. The scattering distances have a mean value around 5 Å in both left and right dissociation with a spread of ±3 Å. This is comparable to the average recoil distances observed for iodine atoms dissociated from diiodo-benzene on Cu(110) along different directions of the surface.[11] The scattering angles have a broad spread of about ±60° for the left dissociation and ±30° for the right dissociation.

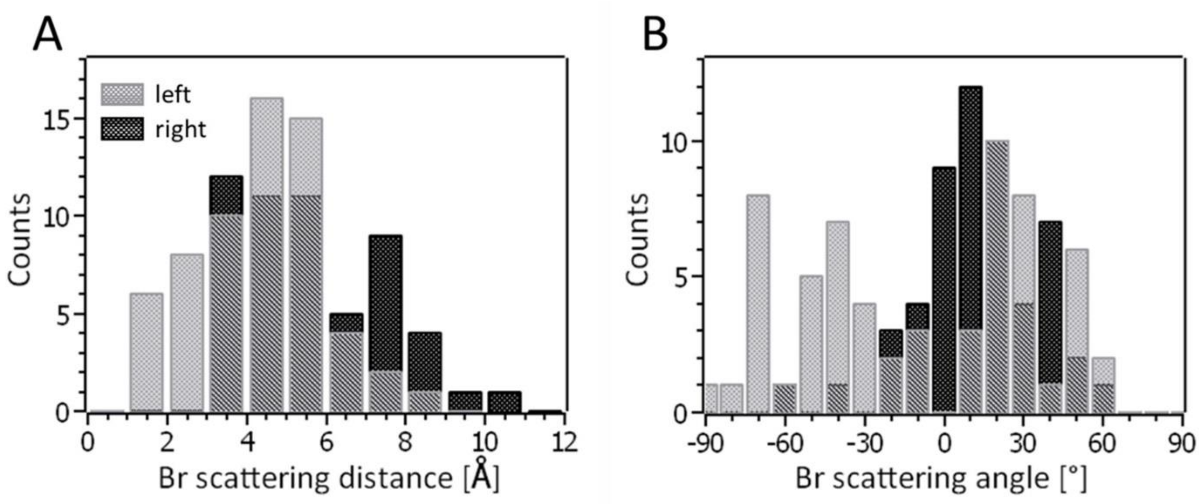

Figure S11. *Histogram of the distribution of scattering angles (A), and scattering distances (B) of the Br atoms after C-Br bond dissociation; grey for the left dissociation and black for the right one.*

12. Colour coding according to BTF orientation

We correlated the Br and BTF positions with the respective orientations of the BTF fragment by colour-coding the data for the left dissociation (Fig. S12). It can be seen that positions of Br atoms are widely distributed across different angles – regardless of the BTF orientation (orange, green, and grey datapoints, and relative histogram columns). Thus, assuming that the Br is ejected along the prior bond direction and excluding overly complex dynamics (where the molecule undergoes rotation both before and after bond dissociation), we find no correlation, pointing to the interaction of the Br atoms with the Ag(111) lattice as the cause of their broad distribution in angle and distance. This is in agreement with previous works, which have studied the recoil of dissociated halogen atoms on the corrugated Cu(110) surface, and explain the dynamics as being significantly influenced by the halogen atom collisions with surface atoms, resulting in preferential motion along the minimum-energy-barrier pathways along or across copper rows.[11,12] Our results on the flat Ag(111) surface suggest that Br atoms are randomly deflected by interactions with surface silver atoms along no preferential direction. On the other hand, the positions of BTF fragments are related to their orientation, which explains that it is actually a rotation of the molecular fragment that displaces its centre from the origin.

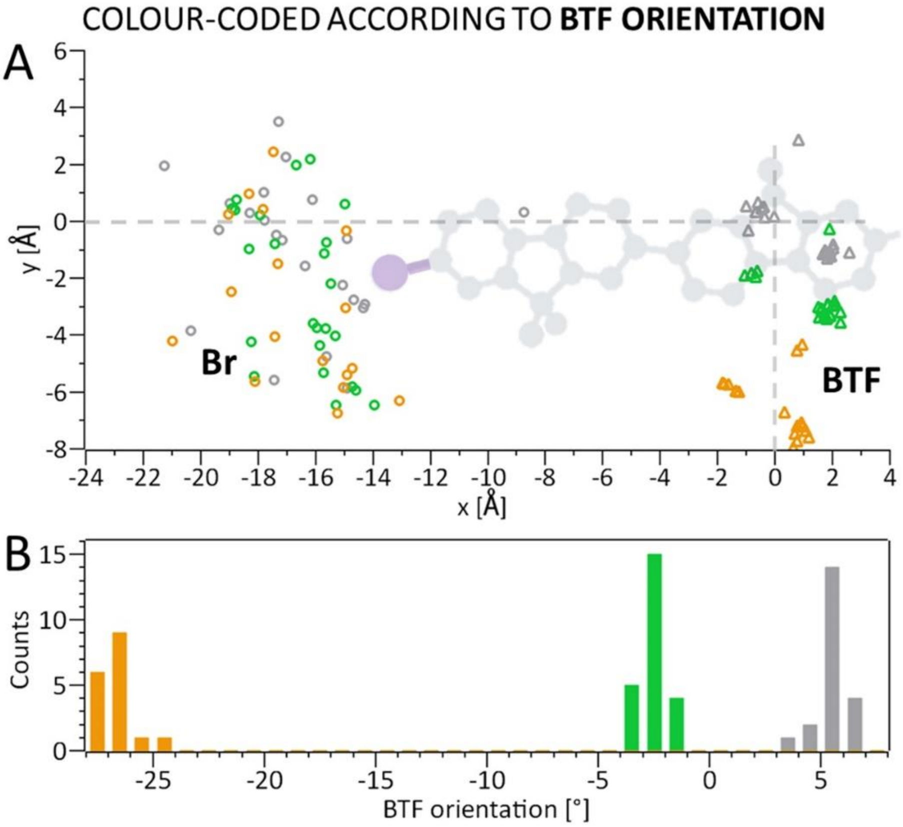

Figure S12. *(A) 2D scatter plot with the circles and the triangles representing the position of the bromine and molecular fragment, respectively (same data as in Fig. 4A of the main text). The intact molecular structure is also shown in its original configuration. (B) Histogram of the BTF orientation after dissociation of the molecular fragment (same as in Fig. 4B). Data points and histogram bars are colour-coded where the colours are relative to the three different peaks of the histogram in (B). No such representation is shown for the right bond dissociations, because there is only one orientation for the BTF (see Fig. 4C).*

13. Rotations of the de-brominated molecular fragment

The experiments show the rotation of the de-brominated molecular fragment after Br dissociation to -2.5° and -27.0° (see Fig. 4B). To explain these values, Fig. S13 shows the two molecular adsorption configurations as described in Fig. S4. For the analysis the data was folded, meaning that experiments done on the molecules in the blue configuration were mirrored and grouped together with the experiments done in the orange configuration. The mirroring is possible due to the symmetry of the molecule (see Fig. S4B).

The re-orientation values of -2.5° and -27.0° could indicate a preferential clockwise rotation since no positive values are present in Fig. 4B. Here we want to specify that if the data are not folded, and the two adsorption configurations are considered separately, then we find preferential rotations in opposite directions, as shown in Fig. S13. Dissociation of the C-Br bonds indicated with a red cross (which are equivalent in their atomic environment) leads to opposite rotations (clockwise and counter-clockwise, respectively) as indicated by black arrows. Importantly, the same final orientations are found (±2.5° and ±27.0°), only in opposite directions.

As explained in the main text, dissociation of the other C-Br bond (not marked with a red cross) does not induce substantial rotation of the molecular fragment.

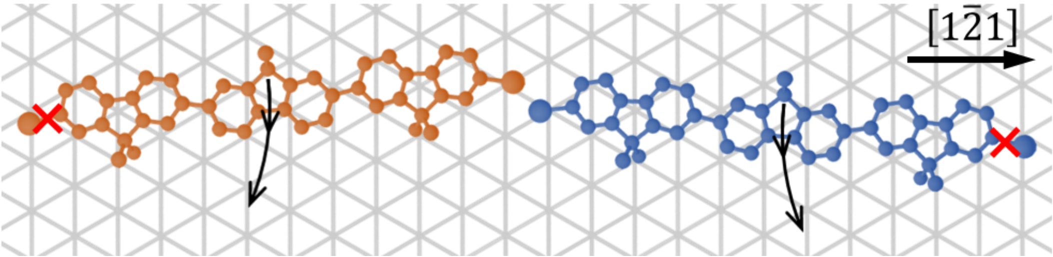

Figure S13. *Scheme of the two adsorption configurations of DBTF on Ag(111), one at +4.5° and one at -4.5° with respect to the* $[1\bar{2}1]$ *symmetry direction, in orange and blue, respectively. The dissociation of the C-Br bond indicated with a red cross (called "left" in the main text) induces rotations in preferred directions, indicated with black arrows.*

14. Determining the pivot point of a de-brominated molecule

To determine the pivot point of a de-brominated molecule, five consecutive STM images obtained following successive lateral manipulations of a single molecule (described in Fig. S6) are summed into one image (Fig. S14). This analysis clearly shows the central pivot point that remains unchanged for all orientations of the molecule, which could not be identified in a single STM image. The position of the pivot point can then be defined with respect to the central fluorene lobe's maximum, which is directly measured. The black arrow indicates the vector that is added to the position of the central fluorene lobe to obtain the position of the pivot point for each BTF fragment.

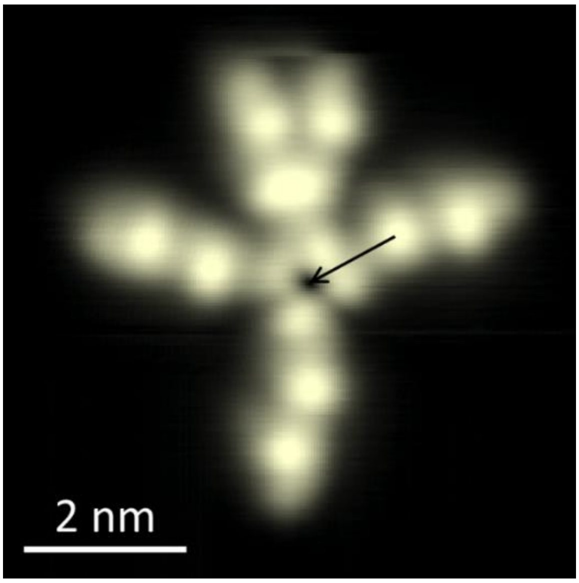

Figure S14. *Sum of the five STM images shown in Fig. S6. The black arrow, which is identified from this analysis, is the vector connecting the central fluorene lobe to the pivot point.*

15. Colour coding according to BTF binding point

The two clusters of the BTF positions (triangles) correspond to the two binding points, respectively. The BTF final orientations (histogram) are independent from the binding point.

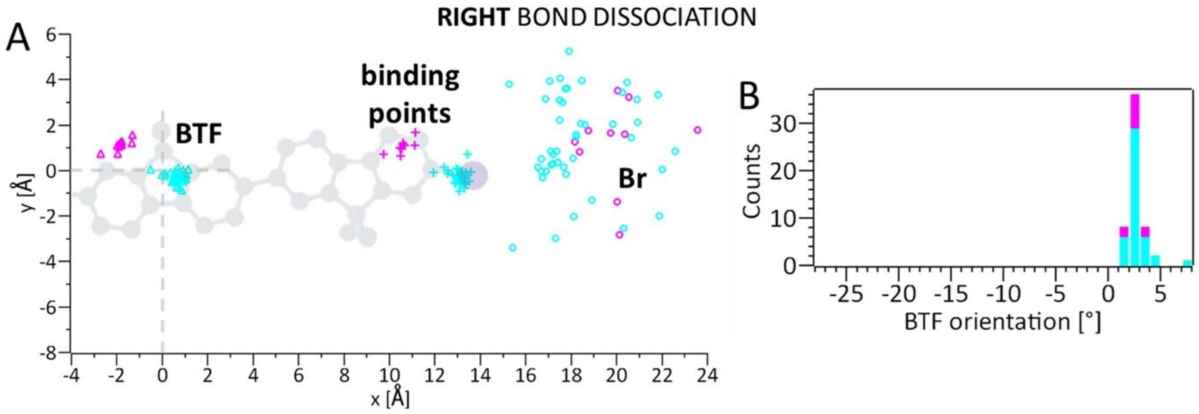

Figure S15. *(A,B) The same scattering plot and histogram of Fig. 4C and D (right dissociations), but colour-coded as in Fig. 5B and C. The colours relate to the two binding points identified by the two clusters of crosses on the right side of Fig. 5A: cyan for the cluster defined as "forward", and magenta for the other defined as "backward". The circles, the triangles and the crosses in B represent the position of the bromine, molecular fragment, and binding point, respectively. The intact molecular structure is also shown in grey. The histogram in B shows the orientation after dissociation of the molecular fragment. The columns for the two cases are added on top of each other.*

16. Calculated adsorption geometry for DBTF

The calculated geometry, corresponding to the experimental adsorption configuration in which the long axis of the DBTF molecules lies at +4.5° to the [12̄1] direction, is given in Fig. S16. The calculated adsorption configuration is in good agreement with the experimentally determined adsorption configuration shown in Fig. S4A (see Section S4 for a detailed description of how the adsorption configuration this was determined). From the top-down view in Fig. S16A the Br atoms (red circles) at the left and right ends of the molecule are seen to adsorb approximately atop the Ag atoms (silver circles). The side-on view (Fig. S16B) shows the heights of these Br atoms above their respective underlying Ag atoms (3.24 Å). The centre of mass of the DBTF molecule sits 3.54 Å above the Ag surface plane, which is slightly higher than the calculated height of 3.22 Å for the most-stable adsorption configuration of benzene on a 3 ML-NaCl/Cu(001) surface.[13] In the same work,[13] the calculated height of benzene above the bare copper surface was 2.26 Å, while the adsorption height of a comparable dihalobenzene on Cu(110) was determined to be 2.36 Å[14]. This suggests that the dimethyl units act to lift the DBTF molecule from the metal surface.

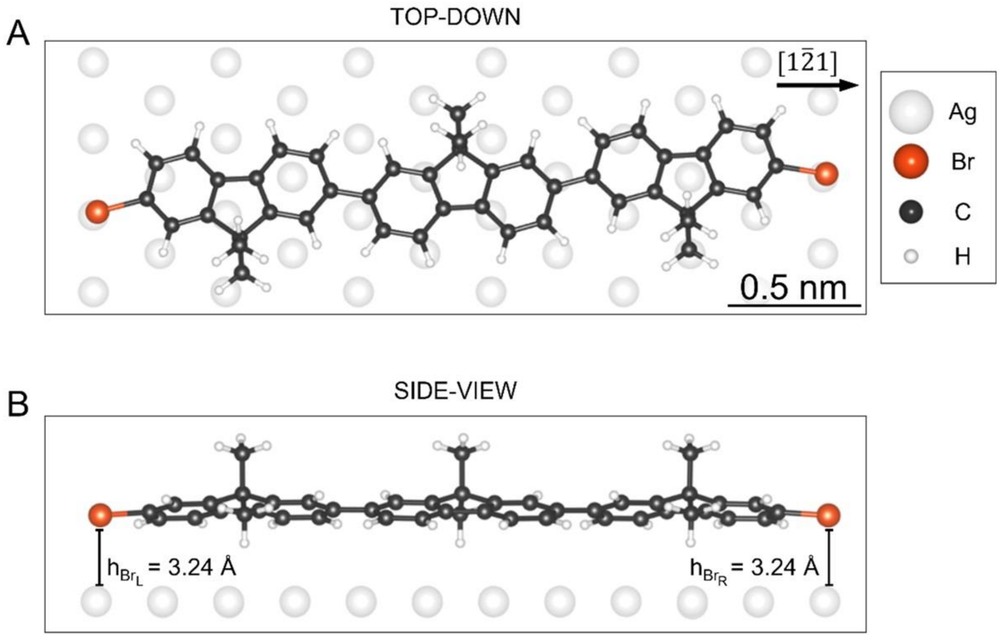

***Figure S16**. (A-B) Calculated adsorption geometry for DBTF on Ag(111) showing (A) top-down and (B) side-on views. The calculated geometry corresponds to the experimental adsorption configuration where the long axis of the DBTF molecules lies at +4.5° to the [12̄1] direction. The calculations were performed by Qifan Chen and Pavel Jelinek (Czech Academy of Sciences).*

References

1. Civita, D. *et al.* Control of long-distance motion of single molecules on a surface. *Science* **370**, 957–960 (2020).
2. Endo, O., Kondoh, H. & Ohta, T. Scanning tunneling microscope study of bromine adsorbed on the Ag(111) surface. *Surf. Sci.* **441**, (1999).
3. Ho, W. Single-molecule chemistry. *J. Chem. Phys.* **117**, 11033–11061 (2002).
4. Riedel, D. Single molecule manipulation at low temperature and laser scanning tunnelling photo-induced processes analysis through time-resolved studies. *J. Phys. Condens. Matter* **22**, (2010).
5. Anggara, K. *et al.* Clocking Surface Reaction by In-Plane Product Rotation. *J. Am. Chem. Soc.* **138**, 7377–7385 (2016).
6. Hla, S. W. & Rieder, K. H. STM Control of Chemical Reactions: Single-Molecule Synthesis. *Annu. Rev. Phys. Chem.* **54**, 307–330 (2003).
7. Jiang, Y., Huan, Q., Fabris, L., Bazan, G. C. & Ho, W. Submolecular control, spectroscopy and imaging of bond-selective chemistry in single functionalized molecules. *Nat. Chem.* **5**, 36–41 (2013).
8. Blum, V. *et al.* Ab initio molecular simulations with numeric atom-centered orbitals. *Comput. Phys. Commun.* **180**, 2175–2196 (2009).
9. Perdew, J. P., Burke, K. & Ernzerhof, M. Generalized Gradient Approximation Made Simple. *Phys. Rev. Lett.* **77**, 3865 (1996).
10. Tkatchenko, A. & Scheffler, M. Accurate molecular van der Waals interactions from ground-state electron density and free-atom reference data. *Phys. Rev. Lett.* **102**, 073005 (2009).
11. Leung, L. *et al.* Retention of Bond Direction in Surface Reaction: A Comparative Study of Variously Aligned p-Dihalobenzenes on Cu(110). *J. Phys. Chem. C* **119**, 26038–26045 (2015).
12. Timm, M. J. *et al.* Contrasting Efficiency of Electron-Induced Reaction at Cu(110) in Aliphatic and Aromatic Bromides. *J. Am. Chem. Soc.* **142**, 9453–9459 (2020).
13. Robledo, M., Pacchioni, G., Martín, F., Alcamí, M. & Díaz-Tendero, S. Adsorption of benzene on Cu(100) and on Cu(100) covered with an ultrathin NaCl film: Molecule-substrate interaction and decoupling. *J. Phys. Chem. C* **119**, 4062–4071 (2015).
14. Panosetti, C. & Hofer, W. A. Adsorption of metadiiodobenzene on Cu(110): A theoretical study. *J. Comput. Chem.* **33**, 1623–1631 (2012).
